# Transformation of Plant to Resource Acquisition Under High Nitrogen Addition Will Reduce Green Roof Ecosystem Functioning

**DOI:** 10.3389/fpls.2022.894782

**Published:** 2022-05-17

**Authors:** Qinze Zhang, Guang Hao, Meiyang Li, Longqin Li, Binyue Kang, Nan Yang, Hongyuan Li

**Affiliations:** ^1^College of Environmental Science and Engineering, Nankai University, Tianjin, China; ^2^School of Life Sciences, North China University of Science and Technology, Tangshan, China

**Keywords:** resource acquisition strategy, green roof, nitrogen deposition, plant functional traits, trait plasticity, ecosystem functioning

## Abstract

Ecosystem engineering, such as green roof, provides numerous key ecosystem functions dependent on both plants and environmental changes. In the recent years, global nitrogen (N) deposition has become a hot topic with the intensification of anthropogenic disturbance. However, the response of green roof ecosystems to N deposition is still not clear. To explore the effects of N addition on plant ecological strategy and ecosystem functioning (biomass), we conducted a 3-month N addition simulation experiment using 12 common green roof species from different growth forms on an extensive green roof in Tianjin, China. The experiment included three different N addition treatments (0, 3.5, and 10.5 gN m^–2^ year^–1^). We found that plants with the resource-acquisitive strategy were more suitable to survive in a high N environment, since both aboveground and belowground traits exhibited synergistic effects. Moreover, N addition indirectly decreased plant biomass, indicating that ecosystem functioning was impaired. We highlight that there is a trade-off between the survival of green roof species and keeping the ecosystem functioning well in the future N deposition. Meanwhile, these findings also provide insights into how green roof species respond to global climate change and offer important information for better managing and protecting similar ecosystem engineering in the background of high N deposition.

## Introduction

Global climate changes and human disturbances are destroying terrestrial ecosystem processes and functioning, especially in urban areas (Grimm et al., 2008; Radhi et al., 2013). Due to the fast development of urbanization, the areas available for urban greening continue to decrease, coupled with the expansion of population in cities, resulting in a growing number of ecological and environmental problems (Davidson, 2009). Ecosystem engineering, such as green roof, can mitigate the adverse effects of urbanization and provide numerous key ecosystem functions (e.g., stormwater management, reduce heat transfer through building roofs, provide habitat for heterotrophs, and improve the air quality, Lundholm, 2015; Lundholm and Williams, 2015; Nagase and Koyama, 2020). Thus, it has become an effective measure to increase the greening areas and improve the quality of the ecological environment. Considering that global climate changes may affect the survival of species, they will inevitably impact the green roof functions (Du et al., 2018). However, it remains unclear how global climate changes affect these processes in green roofs, which also limits the understanding of the role of green roof ecosystems.

Due to the increase of atmospheric reactive nitrogen (N) caused by anthropogenic disturbance, global N deposition has received considerable attention in the recent years, especially in cities, which have become the hot areas of N deposition (Galloway et al., 2004; Davidson, 2009). Increased N deposition has profound consequences for natural and anthropogenic ecosystems. For example, studies based on natural ecosystems have found that moderate N addition would improve the ability of plants to utilize various forms of N for their own photosynthesis and accumulation of organic compounds, which promoted plant growth and biomass, as well as contributed to productivity and ecosystem functioning (Bauer et al., 2004; Ren et al., 2019). On the other hand, excessive N deposition would lead to “N saturation” of ecosystems, increasing the other nutrients loss, and considerably threatening the normal growth of plants and the balance of ecosystems, especially in urban areas (Liu et al., 2010). However, there are still few studies examined the response of green roof ecosystems to N deposition so far. A better understanding of how plants respond to N deposition in green roof ecosystems will provide important information for selecting suitable species, improving sustainable ecosystem engineering, developing greening policies, and getting multiple social, economic, and environmental benefits (Du et al., 2018; Shafique et al., 2018).

Plant functional traits can reflect the plant resource utilization ability, adaptability to environmental changes, and trade-offs among different function strategies (Cornelissen et al., 2003; Lundholm and Williams, 2015). They are often used to explain the underlying causal direction between environmental change and various ecological processes of plants (Lundholm, 2015). Meanwhile, species can be categorized based on the effects of plant functional traits on ecological processes so as to achieve the purpose of species screening for green roofs (Nagase and Koyama, 2020). To resist harsh roof conditions (high winds, drought, large temperature fluctuations, and low substrate water availability, Speak et al., 2012), the plants with conservative traits, such as small-stature succulent plants with thick leaves, seem to be reliable and provide better ecological functions in green roofs (Zhang et al., 2020). Most experiments based on N addition in natural ecosystems found that plant functional traits could respond quickly to the changes in N (Trocha et al., 2017), and plants shifted toward resource acquisition strategy (acquire and use nutrients quickly) with the increase of N availability. Considering that the aboveground and belowground parts of plants face different selection pressures, resource acquisition strategies of aboveground and belowground traits may be different and will change with local environments (Hu et al., 2019). Therefore, it is still controversial whether the responses of aboveground and belowground traits to N deposition are consistent in the green roof ecosystem, which may be largely related to environmental factors and remains to be further explored (Li et al., 2019; Hu et al., 2021). Moreover, trait plasticity is a vital indicator for plants to adapt to varied environments by modifying plant structure and function in response to stress, disturbance, or inputs from environments (Matesanz et al., 2010). Generally, trait plasticity tends to be higher in favorable environments, promoting species to optimize resource acquisition to improve their fitness, while it is lower in stress habitats due to the environmental filtering (Ryser and Eek, 2000; Portsmuth and Niinemets, 2007). For example, with the increase of N and phosphorus (P) supply, *Lycium Ruthenicum* improved the plasticity index of specific root length, which could better access to nutrients and adapt to these environments (Li et al., 2021). Thus, to get a comprehensive understanding of the mechanisms underlying species’ response to environmental changes, it is necessary to explore the response of plant traits and trait plasticity to N deposition in green roofs.

Most studies have suggested that plant biomass is directly related to multiple ecological processes and can be used as a proxy for essential ecosystem functions (Costa et al., 2012; Bongaarts, 2019; Farrell et al., 2021). Likewise, for green roof ecosystems, numerous studies have demonstrated that biomass is positively associated with ecosystem functioning and multifunctionality (Lundholm, 2015; Xie et al., 2018). Previous studies found that excessive and long-term N deposition in urban areas might cause soil acidification, decreasing the availability of soil resources and stability of plant communities (Lu et al., 2014), seriously affecting plant biomass and ecosystem functions. Moreover, green roof ecosystem is vulnerable to the environmental changes because of relatively poor resilience and stability (Lundholm et al., 2015; Zhang et al., 2020). Thus, it is crucial to explore the effects of N deposition on plant biomass and ecosystem functioning in green roof ecosystems to better protect and manage similar ecosystems.

In this study, we attempted to explore the effects of N addition on plant ecological strategy and ecosystem functioning (biomass) in a green roof ecosystem. Here, we selected 12 common green roof plants in Tianjin and assessed the effects of N addition on aboveground and belowground traits, trait plasticity, and biomass of plants. The study focused on three hypotheses: (a) N addition would promote the transformation of plant ecological strategy from resource conservation to resource acquisition, and the aboveground and belowground traits of plants showed similar changes to N addition; (b) N addition would inhibit the trait plasticity of plants and decrease their adaptability to the environment; (c) high N addition would inhibit the biomass of plants, thereby reducing green roof ecosystem functioning.

## Materials and Methods

The experimental site was on the roof of a four-story building (approximately 10 m aboveground) at the College of Environmental Science and Engineering in Nankai University, Tianjin, China (latitude 38.9878° North, longitude 117.3312° East). The annual average temperature in this region is 13.8 °C, the annual average precipitation is 704.5 mm, and the annual average relative humidity is 59%. In the recent years, N deposition rate has been increasing continuously in Tianjin, and the current average level of atmospheric N deposition is up to 35 kgN ha^–1^ year^–1^ (Yu et al., 2019).

### The Experimental Design

We customized twelve wooden modules (120 cm × 120 cm × 30 cm), and each module was divided into nine small pots (30 cm × 30 cm × 30 cm), composed of a growing medium layer, a filter membrane containing a piece of a non-woven geotextile, a drainage system of HDPE (high-density polyethylene) drainage panels, and a root barrier layer including HDPE membranes, totally 108 pots (Supplementary Figure 1). In this study, the substrate employed was made up of 40% pumice, 35% sand, 15% peat, and 10% vermiculite by volume (Zhang et al., 2020; all these materials from Xuebin Horticulture Ltd., in Tianjin), and substrate depth of 15 cm was chosen.

On 23 May 2021, twelve common green roof species from different growth forms in Tianjin (Supplementary Table 1) were grown. We randomly assigned twelve wooden modules into three N addition groups (Supplementary Figure 1). Within each group, one seedling of twelve species was randomly assigned to be planted into each of the 36 pots, with 3 replicates for each species, respectively. Pots were irrigated every day for the first 2 weeks and every 2 days for the next 2 weeks. Seedlings that died during this period were replanted in time. After the first month, we tried not to water the roof until a week when it did not rain. At the end of the acclimation period, the three groups were randomized to one of the three N treatments, including the control (no N addition), normal N addition (3.5 gN m^–2^ year^–1^), and high N addition (10.5 gN m^–2^ year^–1^). For the two N addition groups, an aqueous solution of NH_4_NO_3_ was applied one time a month from July to September (18 July, 26 August, and 21 September, respectively). Meanwhile, the control treatment received the same amount of water every time.

### Plant Sampling and Trait Measurement

**FIGURE 1:**
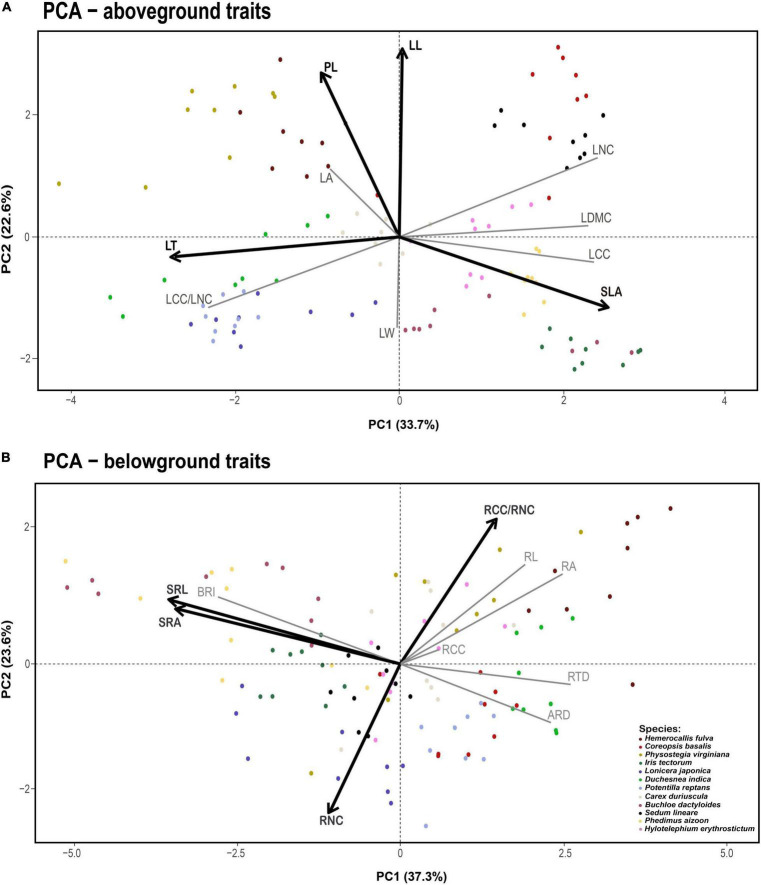
Principal component analysis for aboveground **(A)** and belowground traits **(B)**. Vectors represent the relative contribution of each plant trait to the axis. The two variables that load most strongly on each axis are shown in dark black lines, with the remaining variables shown in light gray. Dots of different colors denote the different species samples. Trait acronyms: LA = leaf area, SLA = specific leaf area, LDMC = leaf dry matter content, LL = leaf length, LW = leaf width, LT = leaf thickness, LNC = leaf nitrogen content, LCC = leaf carbon content, LCC/LNC = the ratio between leaf carbon and nitrogen, PL = plant height, SRL = specific root length, RNC = root nitrogen concentration, RCC = root carbon concentration, RCC/RNC = the ratio between root carbon and nitrogen, RTD = root tissue density, RA = root area, SRA = specific root area, BRI = branching intensity, RL = root length, ARD = average root diameter.

A dataset of aboveground and belowground functional traits including leaf, stem, and root was collected for each species under each N addition treatment by standard methods of Pérez-Harguindeguy et al. (2013). These traits were universally acknowledged as the valid indicators of plant ecological strategies for acquiring, using, and preserving resources such as nutrients, light, and water (Maire et al., 2012; Garnier et al., 2017; Figure 1 depicted trait names and abbreviations; Supplementary Table 2 described the link between the measured traits and their associated functions). After the growing season, the plants were harvested on 21 October to measure plant aboveground and belowground functional traits. Before harvesting the whole plant, we measured the height of each plant (PL). Then, three fully expanded, young and healthy, undamaged leaves per plant were selected randomly to scan and measure leaf length (LL), width (LW), and area (LA) immediately using ImageJ software (version 1.51j8; National Institutes of Health, Bethesda, MD, United States). The scanned leaf fresh weight was measured using an analytical balance (ADAM PGL 203). After that, the leaves and the rest of aboveground leaves and stems (culm plus leaf sheath) were oven-dried at 75°C for 48 h to estimate the dry leaf and aboveground weight (She et al., 2016). Then, we calculated specific leaf area (SLA, the ratio of the leaf area to the leaf dry mass) and leaf dry matter content (LDMC, the ratio of leaf dry mass to fresh mass). Leaf thickness (LT) was calculated as 1/(SLA × LDMC). Finally, dry leaves were powdered for the measurement of leaf carbon content (LCC) and leaf nitrogen content (LNC) using an elemental analyzer (Vario MAX C/N-Macro Elemental Analyzer) and then calculated LCC/LNC.

All plant roots were collected from the pots as completely as possible. After cleaning the fresh roots, 108 root samples for the morphological and architectural measurements were scanned at 400 dpi using a scanner (EPSON Perfection V700/V750) and analyzed (WinRhizo root analysis system, Canada) to calculate the total root length (RL), root area (RA), specific root length (SRL), specific root area (SRA), branching intensity (BRI), and average root diameter (ARD). The corresponding root samples were dried at 75°C for 48 h to determine the final belowground biomass (Lundholm, 2015; She et al., 2016) and then calculated root tissue density (RTD). SRL was calculated as the root length per unit dry weight, SRA was calculated as the root area per unit dry weight, BRI was obtained as the ratio of tip counts to the total root length, and RTD was calculated from root dry weight divided by its volume (Comas and Eissenstat, 2009). In a similar way, dry roots were powdered for the measurement of root carbon content (RCC) and root nitrogen content (RNC), using an elemental analyzer and then calculated RCC/RNC. Finally, we calculated the root–shoot ratio (RSR) of each species using the ratio of belowground dry weight to aboveground dry weight.

### Statistical Analyses

To fulfill the Kolmogorov–Smirnov test of normality distribution and Levene’s test of homogeneity of variances, the data of plant biomass (aboveground, belowground, and total biomass) were log-transformed prior to analysis. Then, one-way analysis of variance (ANOVA) was performed to test the differences in plant biomass (aboveground, belowground, and total biomass) among N addition treatment using the SPSS22.0 (IBM SPSS Inc., Chicago, United States). A *p*-value < 0.05 was considered significantly different, and the means were compared using Tukey’s *post hoc* test.

In addition, to assess the shift in the plant traits along with N addition, separate principal component analyses (PCAs) for aboveground and belowground traits were performed to describe variations in plant traits and visualize the trait space occupied by all 12 plant species. Because the first two axes of PCA explain a high proportion of the plant functional traits (Supplementary Tables 3, 4), the scores of these axes can be used as the overall variations in aboveground and belowground traits (PC of traits) and represent the trends of aboveground and belowground traits under N addition gradient. Because RSR did not belong to the aboveground or belowground traits, we conducted a linear regression between RSR and N addition to assess how RSR values change along the N gradient. Furthermore, pairwise relationships between all aboveground and belowground traits were evaluated by Pearson’s correlation analysis and linear regressions.

To quantify the response of the plant traits to N addition, plasticity index (PI) was calculated (Valladares et al., 2000).


$$\text{PI}=\left({\text{T}}_{\text{iMax}}-{\text{T}}_{\text{iMin}}\right)/{\text{T}}_{\text{iMax}}$$

where T_iMax_ and T_iMin_ are the trait maximum and minimum scores of PC1 or PC2 of species i under each N addition treatment, respectively. Thus, we could get trait plasticity (PI_PC1_ and PI_PC2_) for each species under each N addition treatment.

After this, we used functional traits (PC of traits) and trait plasticity (PI_PC_) of each species in each N addition treatment to make a linear regression with N gradient to explore their connections. Meanwhile, to evaluate whether plant functional traits were properly matched with plant biomass at different N addition, we also performed linear regressions between plant biomass and PC of traits and PI_PC_.

Finally, to quantify the relative importance of each predictor (N addition, PC1 and PC2 of aboveground and belowground traits, and PI_PC1_ and PI_PC2_ of aboveground and belowground traits) in influencing the plant biomass (aboveground, belowground, and total biomass), we performed a variance partitioning analysis with full-model predictors.

All these analyses mentioned above were performed using the packages “FactoMineR,” “factoextra,” “variancePartition,” “psych,” and “vegan” in R version 4.1.1 (R Core Team, 2021).

## Results

### Effects of N Addition on Plant Functional Traits and Trait Plasticity

**FIGURE 2:**
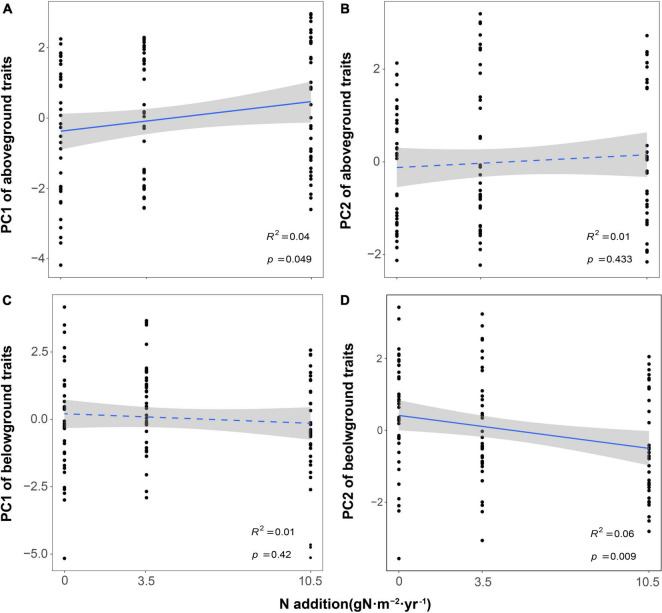
Effects of nitrogen addition on plant functional traits [**(A,B)** for PC of aboveground and **(C,D)** for PC of belowground]. The *R*^2^ (coefficient of determination) and *p*-values are obtained from the linear regression analyses. Shaded areas show 95% confidence interval of the fit test.

The first two independent principal components together explained 56.3 and 60.9% of the total variation for aboveground and belowground traits, respectively (Figure 1). For aboveground traits, the first PCA axis explained 33.7% of the variance and was mainly represented by SLA, LNC, and LT (Leaf Economics Spectrum, LES). We found that PC1 of aboveground traits increased with N addition, which meant the changes in leaf traits reflected a shift from a slow to a fast strategy as N increased (Figure 2A). The second dimension explained an additional 22.6% of the variance, indicating a resource utilization strategy at a trade-off for plant elongation or leaf and stem structure construction. However, N addition did not affect PC2 of aboveground traits (Figure 2B). As for belowground part, the first PCA axis accounted for 37.3% of the variance and described the conservation-acquisition trade-off in root traits (Root Economics Spectrum, RES). N addition had no significant effects on PC1 of belowground traits (Figure 2C), either. The second PCA axis accounted for an extra 23.6% of the variance, indicating N uptake capacity of roots. We observed that PC2 of belowground traits was positively correlated with N addition (Figure 2D). In addition, we found that N addition had no significant effects on the plasticity index of aboveground and belowground traits (Supplementary Figure 2).

**FIGURE 3:**
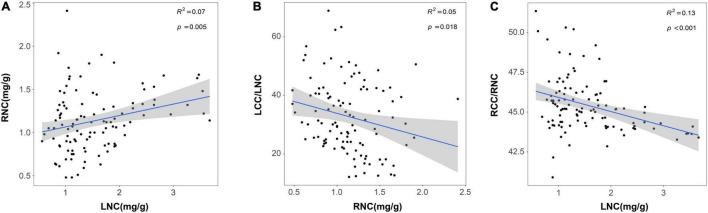
Relationships between LNC and RNC **(A)**; RNC and LCC/LNC **(B)** and LNC and RCC/RNC **(C)**. Trait acronyms: LNC = leaf nitrogen content, LCC/LNC = the ratio between leaf carbon and nitrogen, RNC = root nitrogen concentration, RCC/RNC = the ratio between root carbon and nitrogen. The *R*^2^ (coefficient of determination) and *p*-values are obtained from the linear regression analyses. Shaded areas show 95% confidence interval of the fit test.

Furthermore, we also found a synergy effect between aboveground and belowground traits in response to N addition. The results of Pearson’s correlation analysis showed that LNC was positive with RNC and negative with LCC/LNC and RCC/RNC (Figure 3 and Supplementary Figure 3). SRL and SRA were positively correlated with SLA and both of them were negatively correlated with LT. Likewise, SLA was negatively correlated with RTD and ARD, respectively (Supplementary Figures 3, 4). Moreover, N addition did not affect the plant RSR, and aboveground and belowground biomass had an isometric relationship (Supplementary Table 5 and Supplementary Figure 5), which also proved that the aboveground and belowground biomass allocation strategies of plants did not change.

### Effects of N Addition on Plant Biomass

N addition did not have a significant effect on the whole plant biomass (aboveground, belowground, and total biomass; Supplementary Table 5). The result of variance partitioning analysis also indicated that N addition explained the lowest proportion of variance among all predictors (Figure 6). However, we found that the observed responses of plant biomass to N addition were species-specific-dependent. For example, the aboveground biomass of *Sedum lineare* was significantly increased in high N treatment, but *Buchloe dactyloides* showed the opposite results (Supplementary Figures 6–8).

### Relationships Between Plant Biomass and Functional Traits and Trait Plasticity

**FIGURE 4:**
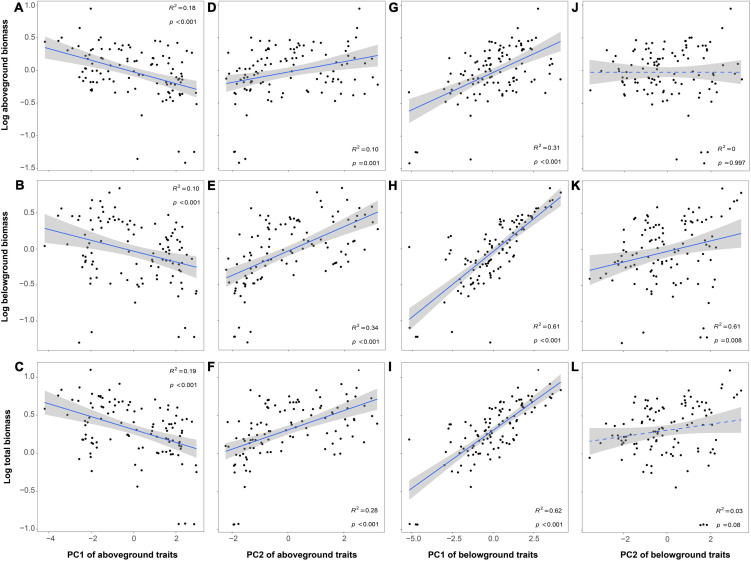
Relationships between plant biomass and plant functional traits [**(A–F)** for PC of aboveground and **(G–L)** for PC of belowground]. The *R*^2^ (coefficient of determination) and *p*-values are obtained from the linear regression analyses. Shaded areas show 95% confidence interval of the fit test.

**FIGURE 5:**
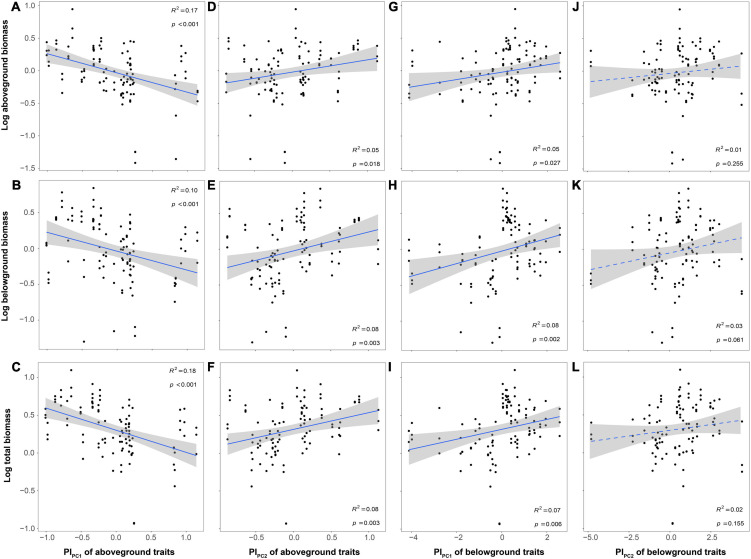
Relationships between plant biomass and trait plasticity [**(A–F)**, for PI_PC_ of aboveground and **(G–L)** for PI_PC_ of belowground]. The *R*^2^ (coefficient of determination) and *p*-values are obtained from the linear regression analyses. Shaded areas show 95% confidence interval of the fit test.

Plant biomass (aboveground, belowground, and total biomass) decreased with PC1 of aboveground traits while increased with PC2 of aboveground traits and PC1 of belowground traits. In addition, only belowground biomass increased with PC2 of belowground traits (Figure 4). The relationship between plant biomass and trait plasticity (PI_PC1_ and PI_PC2_ of aboveground and belowground traits) was similar to the relationships between plant biomass and PC of aboveground and belowground traits (Figure 5).

**FIGURE 6:**
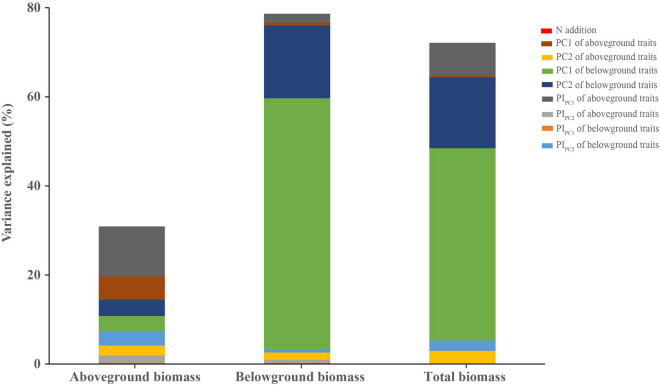
Variance components show the unique portion of variation (percentage of total *R*^2^) explained by each predictor in aboveground, belowground, and total biomass. The predictors used are N addition, plant functional traits (PC1 and PC2 of aboveground and belowground traits), and trait plasticity (PI_PC1_ and PI_PC2_ of aboveground and belowground traits).

By variance component analysis, the sum of unique effects of all predictors explained the 30.9, 78.6, and 72.1% of the total variation in aboveground, belowground, and total biomass, respectively (Figure 6). PI_PC1_ of aboveground traits was the highest variable on aboveground biomass. Next, PC1 of aboveground and belowground traits and PC2 of belowground traits had the similar effects on aboveground biomass (Figure 6). The results obtained for variables from the belowground and total biomass were similar, where the PC1 and PC2 of belowground traits were the first two highest variables and the sum of them could explain 72.7 and 59.1% of the total variation, respectively (Figure 6).

## Discussion

We investigated the effects of N addition on plant resource strategies and ecosystem functioning (biomass) in green roofs. Our findings indicated that plants shifted to resource acquisition strategies with N addition, and the response of these ecological strategies for aboveground and belowground was synergistic. However, we did not find the significant effect of N addition on trait plasticity. In addition, despite N addition did not have significant effects on plant biomass, it could inhibit biomass indirectly by increasing plant resource-acquisitive traits, such as SLA, LNC, and RNC. Therefore, in the context of N deposition, there was a trade-off between the survival of green roof species and keeping the ecosystem functioning. Simultaneously, compared with aboveground traits, root traits played a more important role in the total biomass. In future studies, more attention should be paid to the root growth mechanism of green roof plants under the condition of high N deposition.

### Response of Aboveground and Belowground Traits and Trait Plasticity to N Addition

Our result showed PC1 of aboveground traits increased and PC2 of belowground traits decreased with the increase of N addition, suggesting that both aboveground and belowground traits of plants tended to shift from resource conservation to resource acquisition to adapt to the high N deposition environment. This was similar to the majority of studies in natural ecosystems (Cunningham et al., 1999; Kitajima and Poorter, 2010; Kramer-Walter et al., 2016). For example, SLA and LNC increased and LT decreased with increasing resource availability (such as nutrient and water availability) for the aboveground traits (Cunningham et al., 1999), and RNC was positively correlated with N addition on fertilization experiments for the belowground traits (Helmisaari et al., 2009; Wang P. et al., 2016). N addition could increase soil N availability, which improved the foliar N storage and root absorption efficiency (Butler et al., 2016). Plants with higher LNC tended to have a faster photosynthetic rate, larger SLA, and shorter leaf span (LT is positive with leaf span) in high N deposition (Heberling and Fridley, 2016). Therefore, N addition could promote photosynthetic efficiency and resource acquisition capability which made plants more tolerant to the high N environment. Meanwhile, RNC was thought to be an adequate predictor of root respiration, and roots with high N content increased metabolic activity and nutrient absorption rates (Trocha et al., 2017). With the increase of RNC, more N became available for root uptake and stocked in root tissues, thereby accelerating the cycle of C and N in green roof ecosystems (Nadelhoffer, 2000). In addition, we found that LNC was positive with RNC, both of them were negative with LCC/LNC and RCC/RNC (Figure 3), and N addition did not significantly affect the plant RSR (Supplementary Figure 5). These results also proved that there was a strong coordination between aboveground and belowground traits of roof plants in the direction of resource acquisition strategy in the high N deposition environment (Freschet et al., 2010; Hu et al., 2019).

However, there were no correlations between N addition and PC2 of aboveground traits and PC1 of belowground traits. PC2 of aboveground traits described a trade-off for plant elongation or leaf and stem structure construction (Weiner and Thomas, 1986), playing an important role in determining light interception. This result was similar to Yin et al. (2011), who reported that corn traits related to light interception (such as plant height) were not correlated with N addition. Because of the high winds in the green roof environment (Speak et al., 2012), investing more resources in height to improve light access would incur more costs in construction and maintenance of the stem, resulting a decrease in individual plant fitness and survival rate. Therefore, plants mainly increased LNC and SLA rather than PL in response to N addition. In addition, PC1 of belowground traits described the conservation-acquisition trade-off in the uptake of root resources. Our results showed that N addition did not affect RES strategy, which was different from other studies (Kramer-Walter et al., 2016; Li et al., 2016; Wang et al., 2017). Previous studies found that SRL and SRA showed significantly positive responses to N addition (Li et al., 2016), which was mainly because N could change soil N availability and improve the root absorption efficiency (Butler et al., 2016). However, we did not reach the same conclusion, and the mechanism of N addition on root resource uptake strategies of plants in roof ecosystems is still unclear. We could only deduce that N was not a limiting factor for root resource uptake in our study. In the follow-up roof experiments, further attention and understanding of root system of roof plants are still needed.

Trait plasticity is an important mechanism for plants to optimize resource access and adapt to environmental changes (Wang P. et al., 2016), so we found a positive association between trait plasticity and plant biomass (Figure 5). However, we did not find the significant effect of N addition on trait plasticity, regardless of aboveground or belowground (PI_PC_ of aboveground or belowground). Considering the extreme environmental conditions of the roofs, most of plants we selected were apt to be trait-conservative, and the functional plasticity indexes of these plants were relatively low (Zhang et al., 2020). Therefore, trait plasticity might not be the main strategy for the whole species group to respond to N addition in green roofs. Instead, the roof environment might be the main factor limiting the plasticity of plant traits in green roofs. Species with specific acquisitive traits through environmental filtering were more suitable for growth in this environment (Maire et al., 2012).

### Response of Plant Biomass to N Addition

Plant biomass can represent multiple key ecosystem functions in green roof ecosystems (Xie et al., 2018; Mayer et al., 2021). For example, plant species with greater root biomass had a greater ability in providing nutrient retention and rainwater capture, which also could indicate a general increase in plant resource demand and water consumption (Nagase and Dunnett, 2012). N is one of the most critical nutrients for managing plant function and ecosystem stability, which can affect plant productivity and biomass for both above and below parts (Hermans et al., 2006). However, our results showed that N addition did not influence plant biomass (Figure 6 and Supplementary Table 5), which was different from other studies (Lee et al., 2017; Mao et al., 2017). There might be owing to three reasons. First, the response of species to N addition was specific, leading to an independent correlation between each species’ biomass and N treatment (Ren et al., 2019). Second, N had both positive and negative effects on plant growth (Lu et al., 2014; Li et al., 2016). Therefore, the overall effect was not significant probably because the positive and negative effects were offset each other. Finally, it might be owing to our short experiment period (only one growing season; Lundholm, 2015), which could not reflect sufficiently the relationship between N addition and plant biomass.

Although N addition did not affect biomass directly, it could decrease plant biomass by mediating plant functional traits. Because of the harsh environment on green roofs (Speak et al., 2012), plants adopting resource conservative strategy had higher biomass and ecosystem functioning (Figure 4). However, N addition indirectly decreased the plant biomass and ecosystem functioning by the shift to resource-acquisitive traits. This might be because N addition broke the stoichiometric ratio of original elements in plant organs, leading to the imbalance of nutrient element relations, which was not conducive to plant growth (Nadelhoffer et al., 1999; Baptist and Aranjuelo, 2012; Liu et al., 2018). For example, N addition increased LNC and RNC, which caused that the C/N ratio dropped (LCC/LNC and RCC/RNC, Figure 3). The changes of C and N circulation indicated the original C-N balance of the plant nutrient system would be broken and eventually caused the decrease in plant biomass (C/N balance hypothesis, Bryant et al., 1983; Wang L. et al., 2016). Moreover, resource-acquisitive species appeared to be more affected by water scarcity than conservative ones, due to their higher water demand to maintain high growth rates (Grime et al., 1997; Fort et al., 2014). In our study, N addition promoted the development of plant traits toward the direction of resource acquisition, which intensified the effects of water shortage on roof plants. Thus, considering the environment of water-deficient in green roofs, this might also be one of the main reasons for limiting the growth and biomass of resource-acquisitive species under high N deposition (Fort et al., 2014; Khan et al., 2020).

In addition, the results of variance partitioning analysis showed that the first two principal components of belowground traits played the most important roles in total biomass (Figure 6), which was in line with the understanding that plants would invest more resources for the root growth to survive under stress environment conditions (Wang P. et al., 2016). Therefore, given that root traits had more influence on the total biomass compared with aboveground traits and N addition, it is important to consider how root traits and environmental changes impact plant selection and ecosystem function in green roofs. Meanwhile, it is also worth noting that our experimental periods are relatively short (only a 3-month N addition simulation experiment). The next important question to explore is whether our results on the response of aboveground and belowground traits and biomass to N deposition can be generalized to a longer time scale.

## Conclusion

Our findings demonstrate that plant ecological strategy shifted from resource conservation to resource acquisition with N addition and the responses of both aboveground and belowground traits of plants were synergistic in green roofs. Therefore, considering the survival of plants, plants with resource acquisition traits (large specific leaf area, high leaf and root N concentration, and thin leaves) were more suitable for roof planting in high N deposition. However, the transformation of green roof plants to resource acquisition as N addition also resulted in the decline of biomass and ecosystem functioning. In the context of future high N deposition, a trade-off between the survival of species and their ecosystem functioning needs to be considered when planning and managing similar ecosystem engineering. Furthermore, the research on root traits should be strengthened in the future conservation of green roof plants. These findings provide insights into how species respond to global climate changes in green roofs and support the possibility of the development of sustainable green roofs in a background of high N deposition.

## Data Availability Statement

The raw data supporting the conclusions of this article will be made available by the authors, without undue reservation.

## Author Contributions

GH, NY, and HL conceived the ideas and devised the methodology. GH, QZ, ML, LL, and BK collected the data. QZ and GH analyzed the data. QZ, GH, and HL led the writing of the manuscript. All authors made significant contributions to the drafts, which were eventually approved for publication.

## Conflict of Interest

The authors declare that the research was conducted in the absence of any commercial or financial relationships that could be construed as a potential conflict of interest.

## Publisher’s Note

All claims expressed in this article are solely those of the authors and do not necessarily represent those of their affiliated organizations, or those of the publisher, the editors and the reviewers. Any product that may be evaluated in this article, or claim that may be made by its manufacturer, is not guaranteed or endorsed by the publisher.
