# Supplementary material for: Transformation of Plant to Resource Acquisition Under High Nitrogen Addition Will Reduce Green Roof Ecosystem Functioning
Source: Front Plant Sci. 2022 May 17;13:894782. doi: 10.3389/fpls.2022.894782 (PMC9157423; doi:10.3389/fpls.2022.894782)
Supplement: Supplementary file 1 [file Data_Sheet_1.docx]

Supplementary data

**Extra information on methods and results**

**belongs to the study:**

**Transformation of Plant to Resource Acquisition under High Nitrogen Addition Will Reduce Green Roof Ecosystem Functioning**

***Qinze Zhang, Guang Hao, Meiyang Li, Longqin Li, Binyue Kang, Nan Yang, Hongyuan Li***

**S1 Additional tables**

**Table S1** The list of plant species in our green roof experiment.

| Growth forms | Family | Species |
| --- | --- | --- |
| Tall forbs | Asphodelaceae | *Hemerocallis fulva* |
|  | Asteraceae | *Coreopsis basalis* |
|  | Lamiaceae | *Physostegia virginiana* |
|  | Iridaceae | *Iris tectorum* |
| Creeping shrubs | Caprifoliaceae | *Lonicera japonica* |
| Creeping forbs | Rosaceae | *Duchesnea indica* |
|  | Rosaceae | *Potentilla reptans* var *sericophylla* |
| Sod-forming graminoids | Cyperaceae | *Carex duriuscula* |
|  | Poaceae | *Buchloe dactyloides* |
| Succulents | Crassulaceae | *Sedum lineare* |
|  | Crassulaceae | *Phedimus aizoon* |
|  | Crassulaceae | *Hylotelephium erythrostictum* |

Notes: The 12 species belonged to five distinct "growth forms", groupings of plants with similar morphologies and growth patterns: tall forbs (broad-leaved herbaceous perennials with erect growth), creeping shrubs (perennials with prostrate woody growth), creeping forbs (herbaceous perennials with relatively broad leaves with prostrate growth), sod-forming graminoids (narrow-leaved herbaceous perennials whose belowground rhizomes result in a dense sod), and succulents (plants that store water in leaves and stems).

**Table S2** List of 20 plant functional traits selected.

| Trait | Unit | Description | Characteristic | Function |
| --- | --- | --- | --- | --- |
| **Aboveground traits** | | | | |
| SLA | cm^2^/g | Specific leaf area | Morphology | Assimilate utilization, light interception, space niche in canopy (Wilson et al., 1999; Rowe et al., 2012) |
| LA | cm^2^ | Leaf area | Morphology | Light interception (Wilson et al., 1999; Maire et al., 2012) |
| LL | cm | Leaf length | Morphology | Light interception, avoidance (Maire et al., 2012) |
| LW | cm | Leaf width | Morphology | Light interception, avoidance (Maire et al., 2012) |
| LT | cm | Leaf thickness | Morphology | Water storage (Garnier et al., 2004; Rowe et al., 2012) |
| LDMC |  | Leaf dry matter content | Morphology | Assimilate utilization, decomposability (Maire et al., 2012) |
| LNC | mol/L | Leaf N content | Chemical composition | Photosynthetic capacity (Yang et al., 2009; Ordoñez et al., 2009) |
| LCC | mol/L | Leaf C content | Chemical composition | Assimilate utilization, leaf architecture (Bryant et al., 1983; Maire et al., 2012) |
| LCC/LNC |  | Leaf C/N ratio | Chemical composition | Leaf growth resource limitation (Wilson, 1988; Wang et al., 2016) |
| PL | cm | Plant height | Morphology | Light interception, avoidance (Walburg et al., 1982; Yin et al., 2011) |
| **Belowground traits** | | | | |
| SRL | cm/g | Specific root length | Morphology | Assimilate utilization, nutrient uptake, space niche in canopy, forage efficiency (Li et al., 2016; Ding et al., 2020) |
| SRA | cm^2^/g | Specific root area | Morphology | Assimilate utilization, nutrient uptake, space niche in canopy (Li et al., 2016) |
| RL | cm | Root length | Morphology | Space niche in soil, nutrient acquisition strategy (Maire et al., 2012) |
| RA | cm^2^ | Root area per soil volume | Morphology | Space niche in soil, nutrient acquisition strategy (Maire et al., 2012) |
| RNC | mol/L | Root N content | Chemical composition | Efficiency of nitrogen transport and utilization (Nadelhoffer et al., 1999; Bauer et al., 2004) |
| RCC | mol/L | Root C content | Chemical composition | Efficiency of carbon transport and utilization, root architecture (Bryant et al., 1983; Maire et al., 2012) |
| RCC/RNC |  | Root C/N ratio | Chemical composition | Root growth resource limitation, soil nutrient resource limitation (Wilson, 1988; Wang et al., 2016) |
| RTD | g/cm^3^ | Root tissue density | Morphology | Assimilate utilization, water transport, resource storage (Maire et al., 2012) |
| BRI | cm^-1^ | Branching intensity | Morphology | Fungal colonization (Ding et al., 2020) |
| ARD | cm | Average root diameter | Morphology | Growth time strategy, life span (Ding et al., 2020) |

**Table S3** Principal component analysis (PCA) of aboveground traits of 12 species. Trait acronyms: LA = Leaf area, SLA = Specific leaf area, LDMC = Leaf dry matter content, LL= Leaf length, LW= Leaf width, LT= Leaf thickness, LNC= Leaf nitrogen content, LCC= Leaf carbon content, LCC/LNC= the ratio between leaf carbon and nitrogen, PL= Plant height. Coordinates on the first two axes are shown (percentage values in brackets at column headings refer to the variation explained by each axis; two variables that load most strongly on each axis are in bold).

| Variables | Axis1 (33.7%) | Axis2 (22.6%) |
| --- | --- | --- |
| PL | -0.280 | **0.812** |
| LL | 0.011 | **0.922** |
| LW | -0.008 | -0.453 |
| LA | -0.252 | 0.340 |
| LDMC | 0.690 | 0.056 |
| LNC | 0.724 | 0.396 |
| LCC | 0.711 | -0.126 |
| SLA | **0.753** | -0.350 |
| LCC/LNC | -0.697 | -0.353 |
| LT | **-0.820** | -0.099 |

**Table S4** Principal component analysis (PCA) of belowground traits of 12 species. Trait acronyms: SRL = Specific root length, RNC = Root nitrogen concentration, RCC= Root carbon concentration, RCC/RNC= the ratio between root carbon and nitrogen, RTD= Root tissue density, RA= Root area, SRA = Specific root area, BRI= Branching intensity; RL= Root length, ARD= Average root diameter. Coordinates on the first two axes are shown (percentage values in brackets at column headings refer to the variation explained by each axis; two variables that load most strongly on each axis are in bold).

| Variables | Axis1(37.3%) | Axis2(23.6%) |
| --- | --- | --- |
| SRL | **-0.896** | 0.356 |
| RTD | 0.666 | -0.114 |
| RNC | -0.274 | **-0.822** |
| RCC | 0.152 | 0.080 |
| RCC/RNC | 0.367 | **0.798** |
| BRI | -0.713 | 0.375 |
| RL | 0.489 | 0.557 |
| RA | 0.635 | 0.503 |
| ARD | 0.589 | -0.328 |
| SRA | **-0.869** | 0.307 |

**Table S5** Effects of N addition on plant biomass (aboveground, belowground and total biomass) and root-shoot ratio of 12 species as a whole.

| Variable | N addition | | | | |
| --- | --- | --- | --- | --- | --- |
|  | Control | Normal | High | *F_2,105_* | *P* |
| Aboveground biomass | 1.22±0.84 | 1.52±1.55 | 1.16±0.80 | 1.104 | 0.335 |
| Belowground biomass | 1.42±1.39 | 1.64±1.47 | 1.19±0.92 | 1.091 | 0.340 |
| Total biomass | 2.62±1.85 | 3.11±2.51 | 2.26±1.46 | 1.662 | 0.195 |
| Root-Shoot ratio | 1.46±1.94 | 1.55±1.61 | 1.24±1.07 | 0.377 | 0.687 |

Country mean values ± SE are given (12 species per N treatment and 3 replicates for each species). *p* < 0.05 mean significant difference (Tukey post-hoc test).

**S2 Additional Figure**

| **Nitrogen addition groups** | | |
| --- | --- | --- |
| **Control**  **(0 gN m^-2^ yr^-1^)** | **Normal N addition**  **(3.5 gN m^-2^ yr^-1^)** | **High N addition**  **(10.5 gN m^-2^ yr^-1^)** |
| **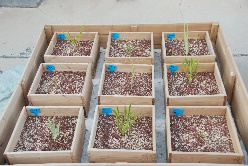** | **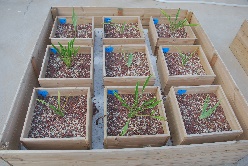** | **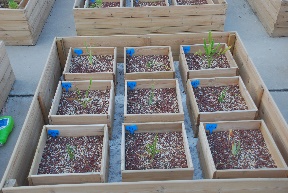** |
| **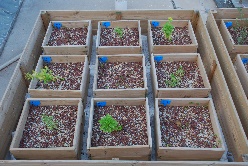** | **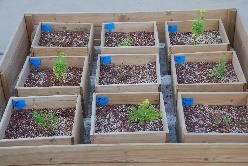** | **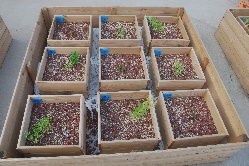** |
| **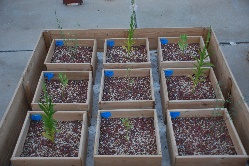** | **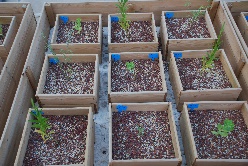** | **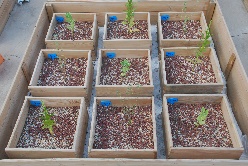** |
| **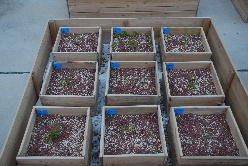** | **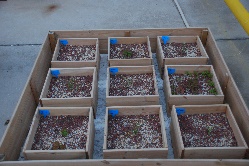** | **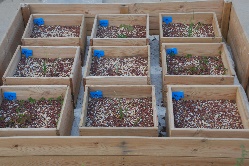** |

**Figure S1** The set of photos for different nitrogen addition groups in this study. There were 12 species under each N addition gradient, and each species had 3 repetitions, each of which occupied a small pot. Meanwhile, each plant was randomly planted in each pot.


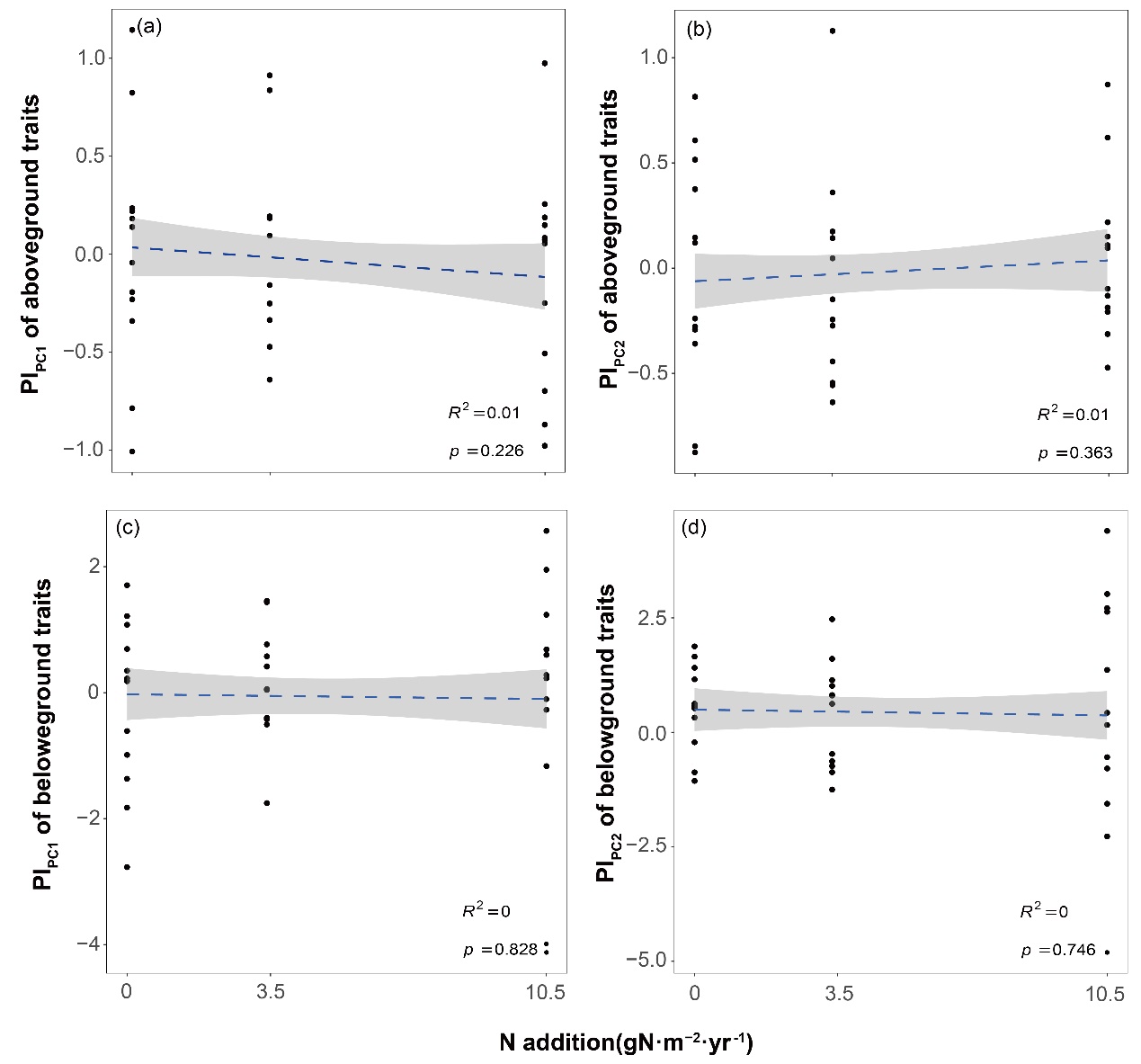


**Figure S2** Effects of nitrogen addition on trait plasticity (a, b for PI_PC_ of aboveground and c, d for PI_PC_ of belowground PC). The *R^2^* (coefficient of determination) and *p*-values are obtained from the linear regression analyses. Shaded areas show the 95% confidence interval of the fit test.


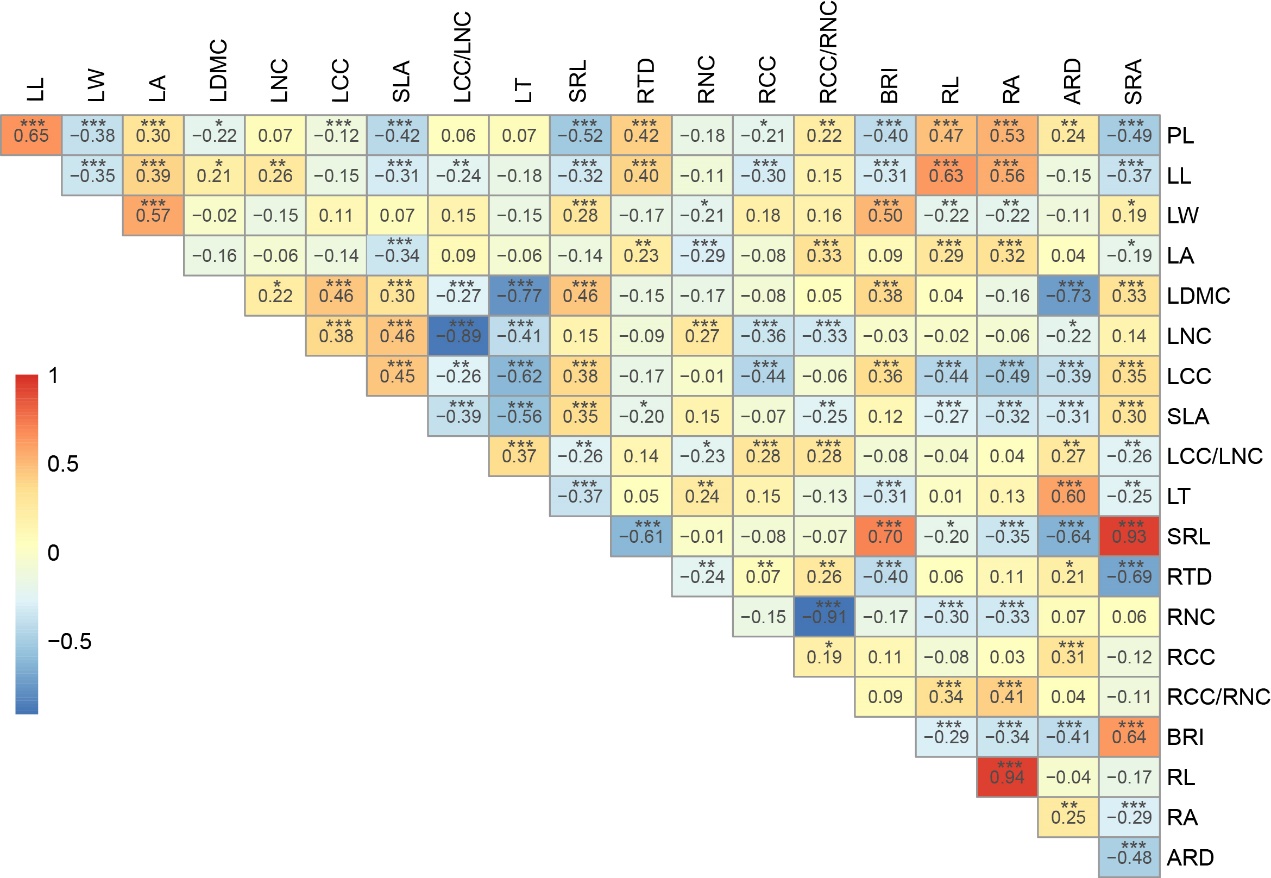


**Figure S3** Correlation matrix of plant functional traits. Person’s correlation coefficient is calculated at sampling grid and test of significance for multiple comparisons between every two traits. Trait acronyms: LA = Leaf area, SLA = Specific leaf area, LDMC = Leaf dry matter content, LL= Leaf length, LW= Leaf width, LT= Leaf thickness, LNC= Leaf nitrogen content, LCC= Leaf carbon content, LCC/LNC= the ratio between leaf carbon and nitrogen, PL= Plant height, SRL = Specific root length, RNC = Root nitrogen concentration, RCC= Root carbon concentration, RCC/RNC= the ratio between root carbon and nitrogen, RTD= Root tissue density, RA= Root area, SRA = Specific root area, BRI= Branching intensity; RL= Root length, ARD= Average root diameter. Symbol *, ** and *** mean significant correlation at the significance level of *p* < 0.05, *p* < 0.01 and *p* < 0.001, respectively.


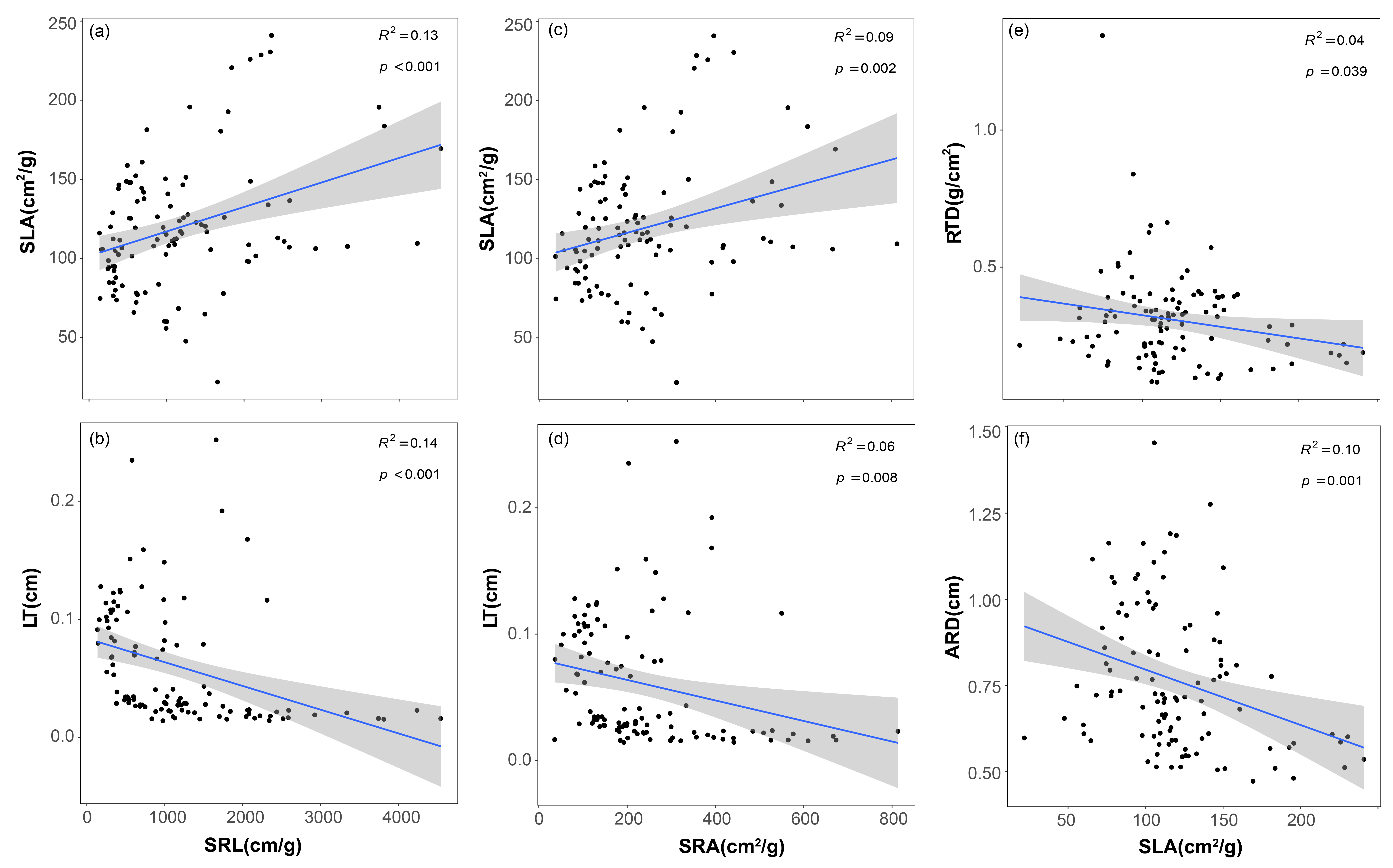


**Figure S4** Relationships between the aboveground and belowground traits from 12 species on the green roof planting. (a, b) relationships between SRL and SLA and LT; (c, d) relationships between SRA and SLA and LT; (e, f) relationships between SLA and RTD and ARD. Trait acronyms: SLA = Specific leaf area, LT= Leaf thickness, RTD= Root tissue density, ARD= Average root diameter, SRL = Specific root length, SRA = Specific root area. The *R^2^* (coefficient of determination) and *p*-values are obtained from the linear regression analyses. Shaded areas show the 95% confidence interval of the fit test.


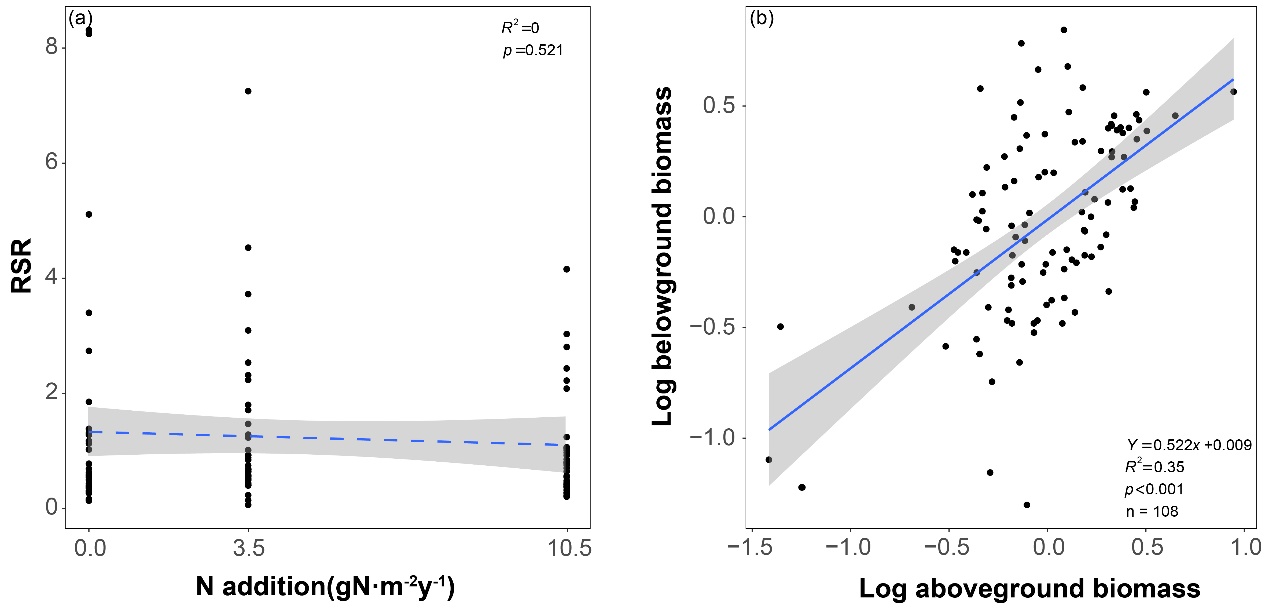


**Figure S5** Effects of N addition on root shoot ratio (a) and relationships between aboveground biomass and belowground biomass (b). Trait acronyms: RSR = Root shoot ratio. The *R^2^* (coefficient of determination) and *p*-values are obtained from the linear regression analyses. Shaded areas show the 95% confidence interval of the fit test.

**
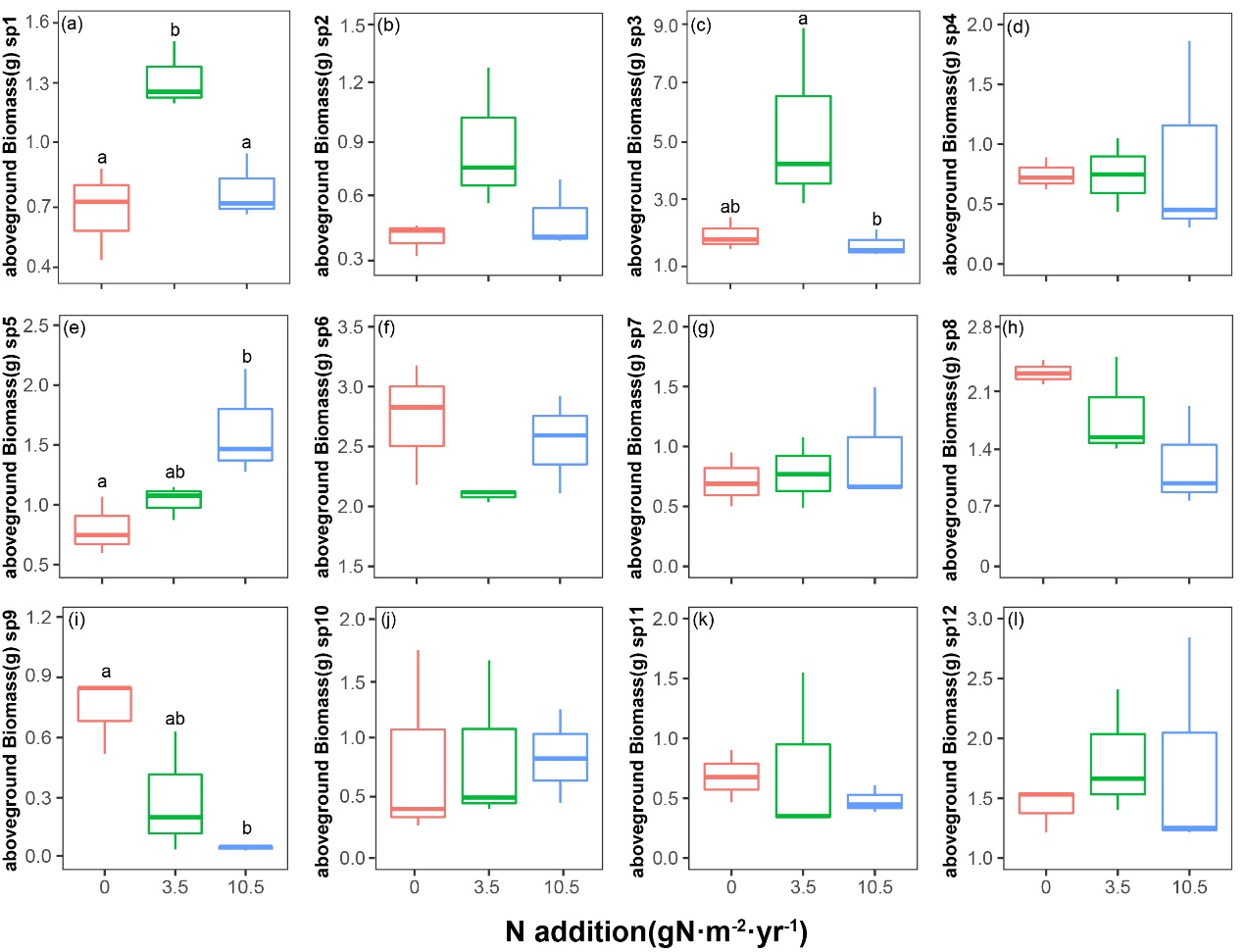
**

**Figure S6** Responses of aboveground biomass to N addition. Different lowercase letters indicate significant difference among N addition treatments (Tukey post-hoc test, *p* < 0.05), while unmarked letters indicate no significant difference. Number of species: sp1= *Iris tectorum*; sp2= *Hemerocallis fulva*; sp3= *Coreopsis basalis*; sp4= *Duchesnea indica*; sp5= *Sedum lineare*; sp6= *Phedimus aizoon*; sp7= *Hylotelephium erythrostictum*; sp8= *Physostegia virginiana*; sp9= *Buchloe dactyloides*; sp10= *Potentilla reptans* var *sericophylla*; sp11= *Carex duriuscula*; sp12= *Lonicera japonica*

**
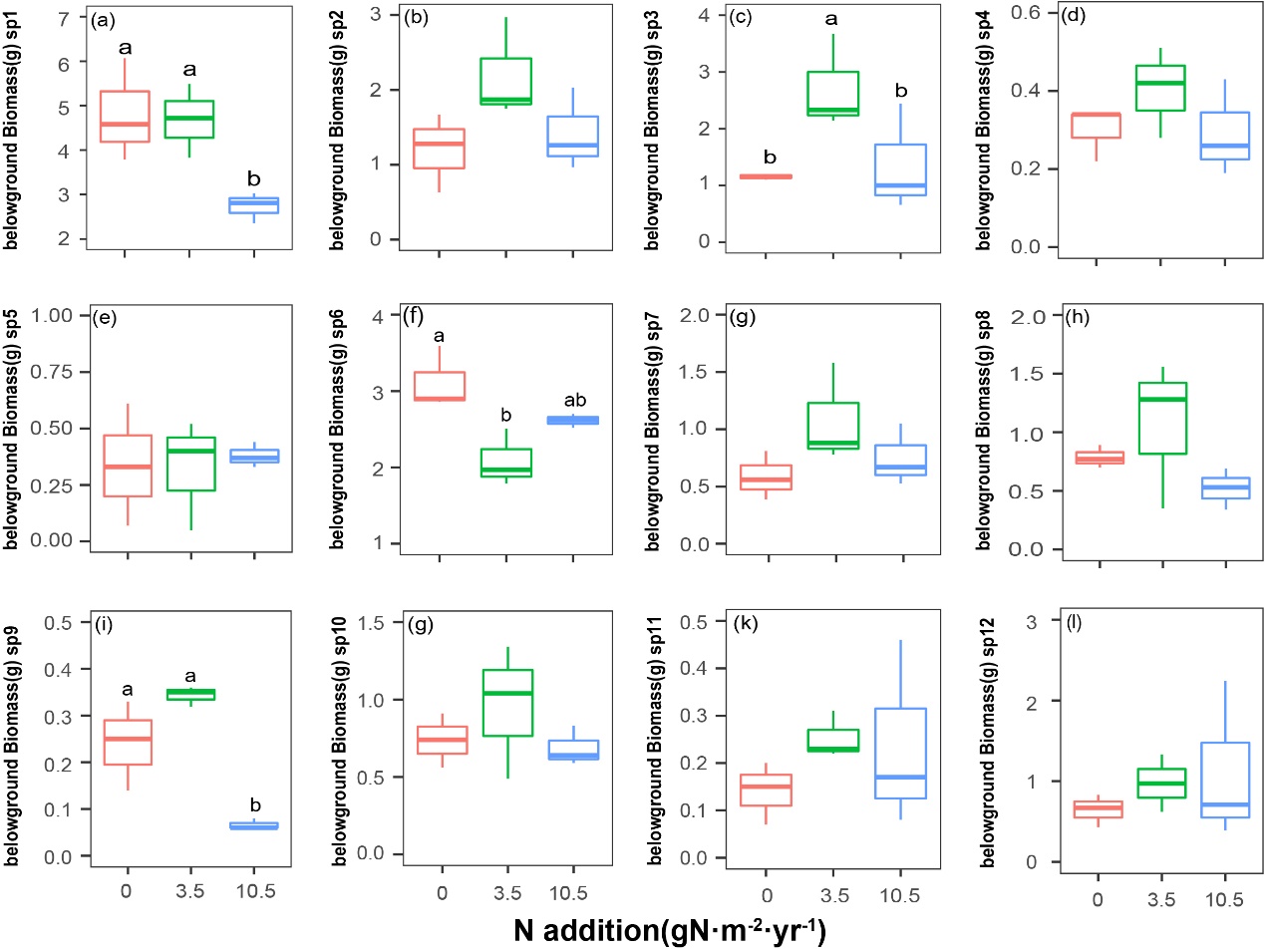
**

**Figure S7** Responses of belowground biomass to N addition. Different lowercase letters indicate significant difference among N addition treatments (Tukey post-hoc test, *p* < 0.05), while unmarked letters indicate no significant difference. Number of species: sp1= *Iris tectorum*; sp2= *Hemerocallis fulva*; sp3= *Coreopsis basalis*; sp4= *Duchesnea indica*; sp5= *Sedum lineare*; sp6= *Phedimus aizoon*; sp7= *Hylotelephium erythrostictum*; sp8= *Physostegia virginiana*; sp9= *Buchloe dactyloides*; sp10= *Potentilla reptans* var *sericophylla*; sp11= *Carex duriuscula*; sp12= *Lonicera japonica*


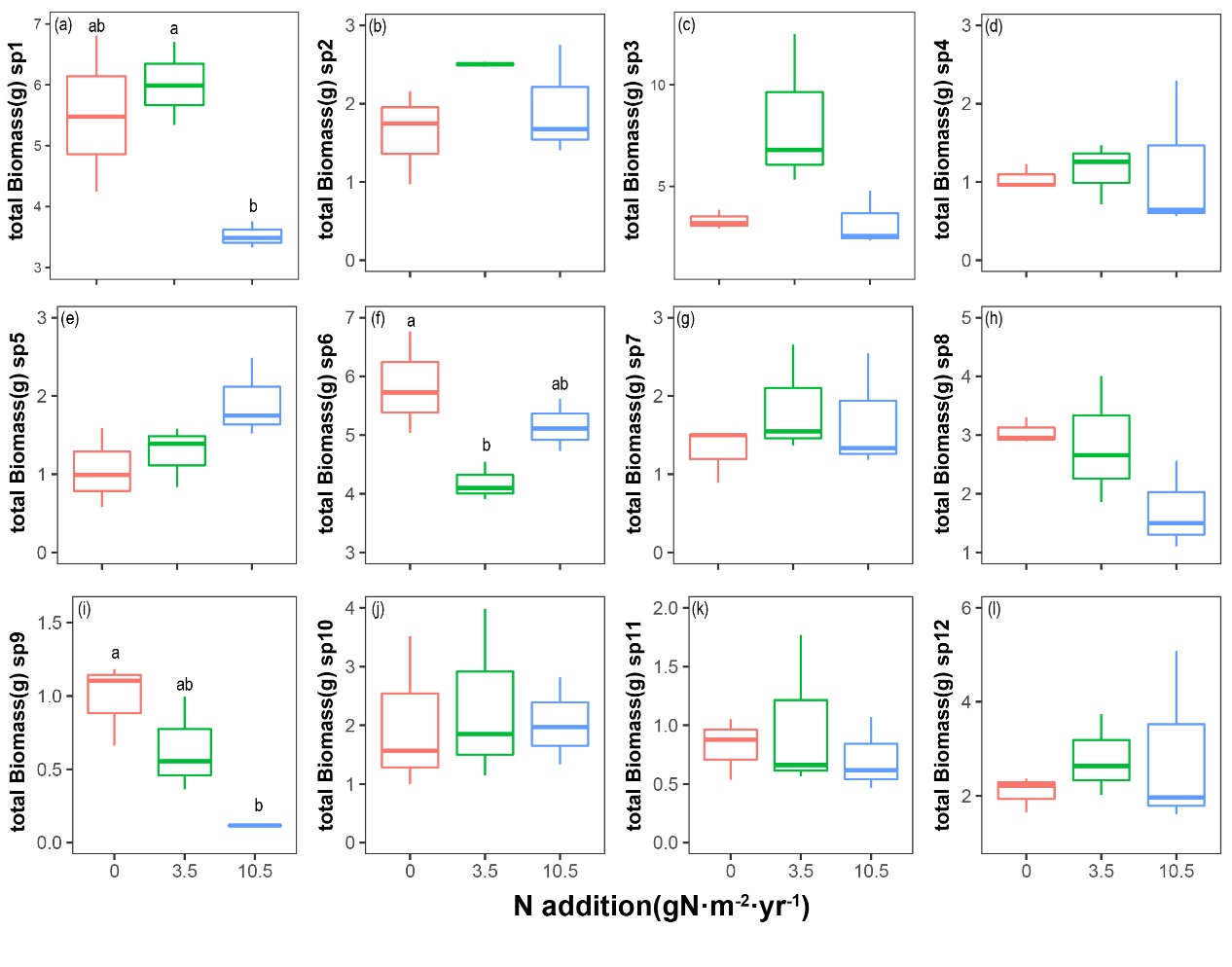


**Figure S8** Responses of total biomass to N addition. Different lowercase letters indicate significant difference among N addition treatments (Tukey post-hoc test, *p* < 0.05), while unmarked letters indicate no significant difference. Number of species: sp1= *Iris tectorum*; sp2= *Hemerocallis fulva*; sp3= *Coreopsis basalis*; sp4= *Duchesnea indica*; sp5= *Sedum lineare*; sp6= *Phedimus aizoon*; sp7= *Hylotelephium erythrostictum*; sp8= *Physostegia virginiana*; sp9= *Buchloe dactyloides*; sp10= *Potentilla reptans* var *sericophylla*; sp11= *Carex duriuscula*; sp12= *Lonicera japonica*

**REFERENCES**

Bauer, G. A., Bazzaz, F. A., Minocha, R., Long, S., Magill, A. H., Aber, J. D., et al. (2004). Effects of chronic N additions on tissue chemistry, photosynthetic capacity, and carbon sequestration potential of a red pine (*Pinus resinosa Ait.*) stand in the NE United States. *Forest Ecol. Manag.* 196, 173-186. doi:10.1016/j.foreco.2004.03.032

Bryant, J. P., Chapin, F. S., and Klein, D. R. (1983). Carbon/nutrient balance of boreal plants in relation to vertebrate herbivory. *Oikos* 40, 357. doi:10.2307/3544308

Ding, J., Kong, D., Zhang, Z., Cai, Q., Xiao, J., Liu, Q., et al. (2020). Climate and soil nutrients differentially drive multidimensional fine root traits in ectomycorrhizal‐dominated alpine coniferous forests. *J. Ecol.* 0, 1-13. DOI:10.1111/1365-2745.13407

Garnier, E., Cortez, J., Billès, G., Navas, M. L., Roumet, C., Debussche, M., et al. (2004). Plant functional markers capture ecosystem properties during secondary succession. *Ecology* 85, 2630-2637. doi:10.1890/03-0799

Li, Y., Niu, S., and Yu, G. (2016). Aggravated phosphorus limitation on biomass production under increasing nitrogen loading: a meta-analysis. *Global Change Boil.* 222*,* 934-943. doi:10.1111/gcb.13125

Maire, V., Gross, N., Börger, L., Proulx, R., Wirth, C., Pontes, L. D., et al. (2012). Habitat filtering and niche differentiation jointly explain species relative abundance within grassland communities along fertility and disturbance gradients. *New Phytol.* 1962, 497-509. doi:10.1111/j.1469-8137.2012.04287.x

Nadelhoffer, K. J., Emmett, B. A., Gundersen, P., Kjønaas, O. J., Koopmans, C. J., Schleppi, P., et al. (1999). Nitrogen deposition makes a minor contribution to carbon sequestration in temperate forests. *Nature* 398, 145-148. doi:10.1038/18205

Ordoñez, J. C., Van, Bodegom, P. M., Witte, J. P. M., Wright, I. J., Reich, P. B., and Aerts, R. (2009). A global study of relationships between leaf traits, climate and soil measures of nutrient fertility. *Global Ecol. Biogeogr.* 18, 137–149. doi:10.1111/J.1466-8238.2008.00441.X

Rowe, D. B., Getter, K. L., and Durhman, A. K., (2012). Effect of green roof media depth on Crassulacean plant succession over seven years. *Landscape Urban Plan.* 104, 310–319. doi:10.1016/J.LANDURBPLAN.2011.11.010

Walburg, G., Bauer, M. E., Daughtry, C. S., and Housley, T. L. (1982). Effects of nitrogen nutrition on the growth, yield, and reflectance characteristics of corn canopies 1. *Agron. J.* 74, 677-688. doi:10.2134/agronj1982.00021962007400040020x

Wang, L., Wang, J., Liu, W. W., Gan, Y., and Wu, Y. (2016a). Biomass Allocation, Compensatory Growth and Internal C/N Balance of Lolium perenne in Response to Defoliation and Light Treatments. *Pol. J. Ecol.* 64, 485-499. doi:10.3161/15052249PJE2016.64.4.004

Wilson, J. B. (1988). A review of evidence on the control of shoot: root ratio. *Ann. Bot.-London* 61, 433-449. doi:10.1093/OXFORDJOURNALS.AOB.A087575

Yang, Y. H., Fang, J. Y., Ji, C. J., and Han, W. X. (2009). Above- and belowground biomass allocation in Tibetan grasslands. *J. Veg. Sci.* 20, 177–184. doi:10.1111/J.1654-1103.2009.05566.X

Yin, X., McClure, M. A., Jaja, N., Tyler, D. D., and Hayes, R. M. (2011). In-season prediction of corn yield using plant height under major production systems. *Agron. J.* 103, 923-929. doi:10.2134/AGRONJ2010.0450
